# A comparison of general surgery training programmes across 11 countries: improving understanding of the experience level of international medical graduates in the UK

**DOI:** 10.1308/rcsann.2024.0086

**Published:** 2024-10-22

**Authors:** KM Spellar, AZ Chacko, C Beaton

**Affiliations:** Royal Devon University Healthcare NHS Foundation Trust, UK

**Keywords:** Training, General surgery, IMG, International medical graduate, Postgraduate training

## Abstract

**Introduction:**

Within the past five years there has been a significant increase in the number of international medical graduates (IMGs) joining the United Kingdom’s (UK) workforce. Having mentors and supervisors who understand the needs of IMGs and clinical and cultural differences in the workplace can benefit in the transition to working in a new country. Improving knowledge of and understanding differences between general surgical training programmes and grades across different countries could therefore aid in the support of IMGs within the UK.

**Methods:**

Data on general surgical training programmes of the top ten countries for the primary medical qualifications of IMGs in the UK were collected to provide comparison with the UK training programme.

**Results:**

The following countries were included: UK, India, Pakistan, Nigeria, Egypt, Ireland, Sudan, Sri Lanka, Romania, Iraq and South Africa. Training programme lengths ranged from 3 to 10 years. Only some training programmes provide additional training and qualification in sub-specialisation in general surgery. Other differences included a requirement for internship/non specialist training prior to training, differences in lengths of time spent in other surgical specialties and a requirement for research.

**Conclusion:**

Understanding the training programmes of other countries may help UK surgeons to understand the prior experience of IMGs and enable them to provide better training and support.

## Introduction

Within the past five years there have been significant changes within the United Kingdom’s (UK) medical workforce, with one notable change being an increase in international medical graduates (IMGs) who have undergone a primary medical qualification (PMQ) outside the UK.^1^ The General Medical Council (GMC) noted a 40% increase in the number of IMG doctors in the past five years, with 50% of doctors who joined the workforce in 2021 being IMGs, in comparison with UK graduates who made up only 39%.^1^ The increase in IMGs has resulted in an increase in both specialty and associate specialist (SAS) and locally employed (LE) doctors, with predictions suggesting that these workforces will soon become the largest on the medical register.^1^

Unfortunately, it has also been noted that, in comparison with UK graduate doctors, IMGs and European Economic Area (EEA) doctors are more likely to leave after joining the UK workforce. Reasons for leaving are complex but have been shown to include dissatisfaction with the role, place of work, National Health Service (NHS) culture, burnout and work-related stress.^2^ An increasing body of qualitative research exploring the integration experiences of IMGs has also noted reasons including a stressful early period, language and communication challenges, differences in culture and medical education, discrimination and belonging.^3–5^ Incentives to aid with integration to the UK include the free GMC half-day induction programme ‘Welcome to UK practice’ and specific trust inductions, but these can be variable in both availability and quality.^6–8^ Having a workplace with supervisors who are familiar with the needs of IMGs and understand the clinical and cultural differences has also been shown to be beneficial in aiding IMGs' transition.^7^

The medical education system within the IMG’s country can be significantly different from the UK, with some systems having an increased emphasis on knowledge and a science-based approach rather than a patient-centred approach with a focus on communication.^5,9^ This difference in medical education systems can result in difficulties for IMGs, because some teaching and assessment methods may be alien to them, making it difficult to properly assess and understand their abilities.^5^ Understanding the medical education system and training within other countries could be beneficial in aiding integration into the UK, particularly for those acting as supervisors, mentors and buddies.

The Royal College of Surgeons of England provides training programmes and specific schemes to aid with IMGs wishing to gain surgical training within the UK, and include the International Surgical Training Programme and the GMC Sponsorship Scheme.^10^ IMGs are also eligible to apply directly for training at foundation level, Core Surgical Training (CST) and registrar training. The expected increase in IMG doctors taking up SAS and LE jobs also means that the number of IMG surgeons within the UK will be significant. This paper therefore aims to compare the training programmes for general surgery for the top ten individual PMQ countries for the IMG workforce in the UK with the UK training system, with the intention of providing information to UK general surgeons working with newly starting IMGs, to expand understanding of the IMGs' level of training and experience and to support these doctors’ progress within the UK.

## Methods

Data for the top ten individual PMQ countries registered with a licence to practise in the UK were collected from publicly available data on the GMC medical register. All doctors with a licence to practise were considered regardless of role, therefore this included trainee doctors, consultant doctors, LE and SAS. Doctors coming from EEA countries were also included within the data. Recent changes have been made for EEA graduates because of Brexit, resulting in EEA doctors no longer benefiting from automatic recognition of professional qualifications within the UK, so it was felt these doctors should not be segregated in a separate category from IMGs.^11^

Publicly available data from official governing bodies, online website resources and relevant publications regarding medical education were searched to document the training pathway and training requirements within each of the top ten countries, and the UK.

## Results

**Figure 1:**
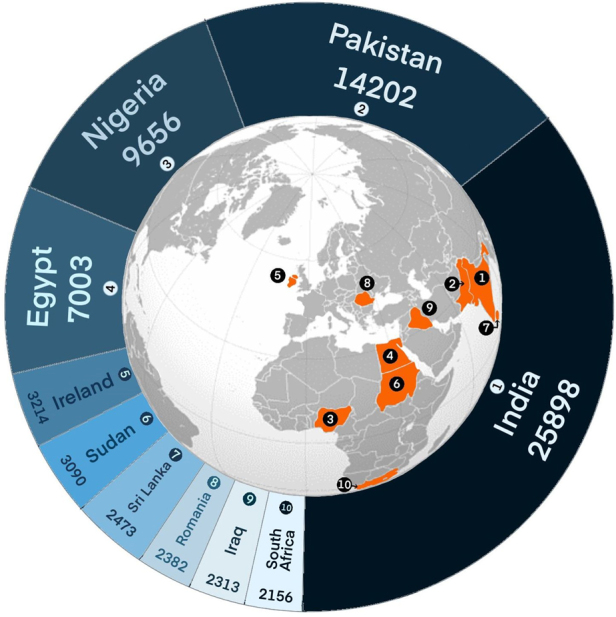
Data extracted from the General Medical Council (GMC) register demonstrating the top ten countries from which international medical graduate doctors working in the United Kingdom have graduated as of 3 June 2023. Please note the GMC has not distinguished between Sudan and South Sudan.

The top ten PMQ countries registered with a full licence to practise in the UK and the number of UK graduate doctors were documented from the GMC medical register as of 3 June 2023.^12^ Doctors working in England, Scotland, Wales and Northern Ireland were included, as were all grades of doctor. Doctors with a provisional or temporary registration were not included. The specific number of doctors is documented in Figure 1. An overview of the various training programmes is given in Table 1 including the UK training programme as a comparison. Details regarding the curriculum for general surgery for each country is given in Appendix 1 (available online). Information documenting the various examinations required by different countries is given in Appendix 2 (available online).

**Table 1:** Comparison of the different training programmes for general surgery among 11 different countries

Country	Stage of training	Duration (minimum)	Entry requirements	Selection process	Logbook requirement (Y/N)	Research requirement	Exit requirements	Total duration post medical school (minimum)
UK	Foundation training	2 years	PMQ. Provisional GMC licence	National application			Evidence of achievement of curriculum. Full GMC registration, completion of 24 months training	
	Core Surgical Training	2 years	Foundation competencies, MSRA exam, self-assessment scoring system	National application	Y	No	MRCS. Evidence of achievement of curriculum with logbook	
	Specialty Registrar Training	6 years	MRCS and CST competencies	National application	Y	Must show evidence of meeting learning outcomes in the curriculum for research	FRCS. Evidence of achievement of curriculum with logbook	10 years to be a sub-specialty general surgeon
India	CRMI	1 year	Provisional Medical Council of India registration. Completed as part of final year of medical school				Evidence of achievement of curriculum	
	Masters of Surgery, residency	3 years	PMQ. Entrance examination (various)	Direct application to university	Y	Thesis	Theory and practical exams, thesis, logbook	3 years to work independently as a general surgeon
	Magister Churugiae (MCh), senior residency	2–3 years	Master of Surgery, Entrance exam (various)	Direct application to university	Y	Thesis	Theory and practical exams, thesis, logbook	5–6 years to be a sub- specialist
or	CRMI	1 year	Provisional Medical Council of India registration. Completed as part of final year of medical school				Logbook evidence of achievement of curriculum	
	DNB	3 years	Entrance examination (NBE)	National Application	Y	Dissertation/thesis	Final theory examination. Dissertation/thesis, logbook	3 years to work independently as a general surgeon
	Doctorate of National Board – Surgical gastroenterology	3 years	Master of Surgery/DNB. Entrance examination – NEET-SS	National application	Y	Dissertation/thesis. Attendance of workshops organised by national board of examinations	Dissertation/thesis, logbook. Final theory and practical examination	6 years to work independently as a specialist gastroenterology surgery
Pakistan	Internship	1 year	PMQ approved by the PM&DC. Provisional registration with PMC or PM&DC				Completion of a basic life support certificate, passing of the National registration examination	
	First fellowship	4–5 years	Internship, FCPS-1 examination	National application	Y		IMM exam at year 2, FCPS exit exam	5–6 years to be a general surgeon
	Second fellowship	3 years	First fellowship	National application	Y	Dissertation or 2 research papers published or accepted for publication	FCPS-II exam	8–9 years to be a sub-specialist
or	Internship	1 year	PMQ approved by the PM&DC. Provisional registration with PMC or PM&DC				Completion of a basic life support certificate, passing of the National Registration examination	
	MS General Surgery	4 years	Internship	Direct application to university	Y	Thesis	Examinations and thesis	5 years to be a general surgeon
Nigeria	Internship	1 years	PMQ. Provisional licence from the Medical and Dental Council of Nigeria				Pass the final medical and dental council of Nigeria exam, performance reports with an average score >60%	
	General surgery residency training – part 1	2 years	Internship. Primary (entrance exam)	National application	Y		Written and clinical examinations, placement requirements	
	General surgery residency training – part 2	4 years	Membership (part 1 completion)	National application	Y	Execution of a research project/dissertation	Written and clinical examinations, placement requirements	7 years to be a general surgeon
Egypt	Internship	1 year	PMQ				Completion of the Egyptian Medical Licencing examination	
	Masters of Surgery, resident	3 years	Admission interview and internship	Direct application to university	Y	Thesis	Written and clinical examinations, placement requirements and thesis	
	MD in Surgery	3 years	Admission interview. Master of Surgery	Direct application to university	Y	Thesis	Written and clinical examinations, placement requirements and thesis	7 years to be a general surgeon
Or	Internship + compulsory service	2 years	PMQ				Completion of the Egyptian Medical Licencing examination	
	Fellowship of the Egyptian Board Programme	5 years	Compulsory service, maximum age limit, must be a resident doctor within surgery for at least 6 months	National application	Y		Examinations	7.5 years to be a general surgeon
Ireland	Internship	1 year	PMQ. Basic life support or intermediate life support. Intern employment eligibility assessment. Registration with the Medical Council of Ireland	National application			Completion of Intern assessment forms	
	Core Surgical Training	2 years	Good undergraduate academic record, surgical aptitude test, interview	National application	Y	No	MRCS. Evidence of achievement of curriculum with logbook	
	Specialty Registrar Training	6 years	MRCS and CST competencies	National application	Y	Must show evidence of meeting learning outcomes in the curriculum for research	FRCS. Evidence of achievement of curriculum with logbook	9 years to be a sub-specialty general surgeon
Sudan	Internship	1 year	PMQ					
	MD in surgery	5 years	Internship, Interview	National application	Y	Thesis	Thesis, Sudan Medical Specialisation Board examinations	6 years to be a general surgeon
Or	Internship	1 year	PMQ					
	COSECSA membership	2 years	Internship, Interview	International application	Y		Membership examinations MCS (ECSA)	
	COSESCA fellowship	3 years	MCS (ECSA)	International application	Y	Research project and publication of one article in a peer-reviewed scientific journal	FCS Gen (ECSA) examinations	6 years to be a general surgeon
Sri Lanka	Internship	1 year	PMQ				Completion of progress reports	
	SHO	1 year	Internship		Y			
	Surgical MD programme	3 years and 2 months	Internship and SHO experience, selection examination	Direct application to university	Y	Research project and to publish or present an oral/poster presentation as a first author	Placement requirements, final MD examination	5 years to be a general surgeon
	Post MD training programme	3 years	Surgical MD. Interview	Direct application to university	Y	Carry out a research project/dissertation	Placement requirements, exit examination	8 years to be a sub-specialist
Romania	General Surgical Residency	6 years	PMQ. Interview	Direct application to university	Y	Activity in research projects	Portfolio requirement	6 years to be a general surgeon
Iraq	Clinical clerkship	1 years	PMQ					
	SHO (rural and remote cities)	1 years	Clinical clerkship					
	General surgical training – Iraqi or Arabian Board of Medical Specialties	5 years	Clinical clerkship and SHO year		Y	Completion of research project that has been either published or presented	Theoretical and practical examinations	7 years to be a general surgeon
South Africa	Internship	2 years	PMQ				Logbook submission with evaluation of performance for each rotation	
	Community service	1 year	Internship					
	Registrar training	1 year	FCA(SA) primary examination	Direct application to university/hospital	Y		Completion of intermediate examination for the FCS(SA)	
	Registrar training (numbered post)	3 years	Intermediate examination for the FCS(SA)	Direct application to university/hospital	Y	Completion of MMed degree	MMed degree, Final examination for FCS(SA), placement requirements	7 years to be a general surgeon
	Sub-specialty	2 years	FCS(SA)	Direct application to university/hospital	Y		Examinations	9 years to be a general surgeon

COSECSA = College of Surgeons of East, Central and Southern Africa; CRMI = Compulsory Rotating Medical Internship; CST = Core Surgical Training; DNB = Diplomate of National Board in Surgery; ECSA = East, Central and Southern Africa; FCA(SA) = Fellowship of the College of Surgeons of South Africa; FRCS = Fellowship of the Royal College of Surgeons; GMC = General Medical Council; IMM = Intermediate Module Exam; MCS = Member of the College of Surgeons; MD = Medical Doctorate; MMed = Master of Medicine; MRCS = Membership of the Royal College of Surgeons; MSRA = Multi-Specialty Recruitment Assessment; NBE = National Board of Examinations; NEET-SS = National Eligibility cum Entrance Test Super Specialty; PM&DC = Pakistan Medical and Dental Council; PMC = Pakistan Medical Commission; PMQ = primary medical qualification; SHO = Senior House officer; References used to collate data^13–78^

### Foundation training and equivalencies

Medical training within the UK begins with the foundation programme, which doctors are allocated to following medical school. All the included countries, except Romania, have an equivalency to the UK’s foundation programme, typically called an internship. India is the only country that includes an internship year during medical school. An additional comparison of these programmes is given in Table 2.

**Table 2:** Comparison of the UK Foundation Programme with different internship programmes between ten different countries, demonstrating requirement for surgical placements

Country	Name of programme	Duration (years)	Specialties included	Requirement to have experience in a surgical specialty	Awarded at completion
UK	Foundation Programme	2	Various and multiple	No specific requirement	Foundation Programme Certificate of Completion
India	Compulsory Rotating Medical Internship (CRMI)	1	Various and multiple	Yes, 2 months	Permanent Medical Council of India Registration Final medical degree CRMI completion certificate.
Pakistan	Internship/house officer	1	Various and multiple	Yes, 3 months in general surgery	Full registration with Pakistan Medical and Dental Council House job certificate
Nigeria	Internship	1	Various and multiple	Yes, 4 months	Full registration with the Medical and Dental Council of Nigeria
Egypt	Internship	1–2	Various and multiple	Yes, 2 months	Registration with the Ministry of Health and the Egyptian Medical Syndicate
Ireland	Intern Training Programme	1	Various and multiple	Yes, 3 months	Certificate of Experience from the Medical Council of Ireland
Sudan	Internship	1	Up-to-date information unavailable; previously variable and multiple	Yes	Registration with Sudan Medical Council
Sri Lanka	Internship	1	Various (2 different placements)	6 months in either general surgery, obstetrics and gynaecology or paediatric surgery	Full Registration with the Sri Lanka Medical Council Certified Internship Certificate of Experience
Romania	No programme				
Iraq	Clinical clerkship/internship	2	Various and multiple	Yes, at least 3 months	Full registration with Medical Council
South Africa	Internship (and community service)	2 (and 1year community service)	Various and multiple	Yes, 3 months general surgery	Intern Duty Certificate, enabling registration with board to perform community service

References used to collect data^13,54–66^

### Core Surgical Training and equivalencies (Phase 1)

Following completion of foundation training, the next step for doctors in the UK if they wish to train in general surgery is to pursue CST. After completion of CST, the next step is to enter general surgical registrar training. Separate application is required to enter both of these training programmes, with various requirements as displayed in Table 1. Effectively, this splits training into two different phases; a more detailed comparison of CST with specific training components with other phase 1 surgical programmes is made in Table 3.

**Table 3:** Comparison of first phase of general surgical training among 11 different countries*

Country	Name of programme	Duration (years)	Specialties included	Assessments	Mandatory courses	Development of laparoscopic skills included in curriculum
UK	Core Surgical Training	2	Various surgical specialties with4–6 month placements. Some programmes are specialty themed	Mandatory CEX/DOPS for critical skills Trainee self- assessments Multiple consultant reports Surgical logbook MRCS	ATLS or equivalent	Desirable if on general surgical placement
India	Master of Surgery – Residency	3	General surgery with other specialty experience including neurosurgery, urology, plastics and paediatric surgery	Thesis Performance assessments Logbook Theory and oral examinations	No	Yes – Should be able to perform laparoscopic cholecystectomy independently
or	Diplomate of National Board in Surgery	3	ED, ICU, cardiothoracic surgery, neurosurgery, surgical gastroenterology, surgical oncology, paediatric surgery, plastics, genitourinary	Logbook Thesis Formative assessment tests Final summative assessment	No	Yes
Pakistan	First Fellowship	4–5	General surgery, orthopaedics/trauma, cardiovascular surgery, neurosurgery, paediatric surgery, thoracic surgery and urology	Logbook Intermediate module examination	Basic surgical skills Research methodology Biostatistics and dissertation writing, communication skills Advanced cardiac life support BLS ATLS Advanced life support in obstetrics Introduction to computer and internet	Depends upon hospital and curriculum
or	MS General Surgery – see Table 4					
Nigeria	General Surgery Residency Training – Part 1	2	General surgery, trauma, urology, orthopaedic, anaesthetics, specialised surgical specialties	Logbook Membership examination	No	No specific requirements
Egypt	Master of Surgery	3	Variable dependent on the university curriculum	Thesis Logbook	Some curricula require ATLS	Included within some university curricula
or	Egyptian Fellowship – see Table 4					
Ireland	Core Surgical Training	2	First Year rotations in general surgery and another surgical specialty. Second Year spent in chosen specialty	Trainee assessment reports Logbook HFPS and OSS assessment MRCS	OSS course HFPS course	Desirable if on general surgical placement
Sudan	Medical Doctorate in Surgery – Part 1	2	6 months in general surgery, 3, 4-month rotations in other surgical specialties	Logbook Part 1 Sudan Medical Specialisation Board examination	No	No
or	COSECSA membership	2	At least 6 months general surgery, 6 months emergency orthopaedic surgery, plus any other surgical specialty	Logbook Examination MCS cases Completion of at least 6 *Surgery in Africa* journal club modules	Basic surgical skills course Basic surgical science course Approved critical care or trauma course	Should have exposure to laparoscopic procedures
Sri Lanka	Surgical MD Programme	3 (+2 months)	18 months general surgery, 4 months orthopaedics, 4 months ED and critical care, 2 months cancer surgery, 2 months cardiothoracic, 2 months neurosurgery, 2 months paediatric surgery, 2 months urology, 1 month gastrointestinal surgery, 1 month plastic surgery	MD examination OSCE In-service training assessment Research project Portfolio assessment (including logbook)	Basic laparoscopic skills course Professionalism and ethics in medical practice strand	No
Romania	Only 1 phase of training. See Table 4	–	–	–	–	–
Iraq	Only 1 phase of training. See Table 4	–	–	–	–	–
South Africa	Registrar training	1	6 months general surgery, at least 3 months ICU, at least 3 months trauma/emergency surgery	Record of procedures (logbook) Clinical practice rating and evaluation - Portfolio	Basic surgical skills course or the basic and essential surgical skills training course ATLS	No

ATLS = Advanced Trauma Life Support; BLS = Basic Life Support; CEX = clinical evaluation exercise; COSECSA =The College of Surgeons of East, Central and Southern Africa; DOPS = direct observation of procedural skills; ED = emergency department; HFPS = human factors and patient safety; ICU = intensive care unit; MCS = Member of the College of Surgeons; MD = medical doctorate; MRCS = Membership of the Royal College of Surgeons; OSCE = objective structured clinical examination; OSS = operative surgical skills

* Countries where there is only one phase of training have not been included here and are instead documented in Table 4

References used to collect data.^23,24,34,51,66–77^

### General surgery registrar training and equivalencies (Phase 2)

General surgical registrar training within the UK is a 6-year programme and typically includes sub-specialisation within a general surgical field after completion of year 4. India, Pakistan, Ireland, Sri Lanka and South Africa all also offer sub-specialisation. A comparison of phase 2 surgical programmes with the UK programme is made in Table 4. Where a country only has one phase of training, these programmes have been included only in Table 4 and are compared with the UK general surgical registrar training programme. Upon completion of the requirements of the UK general surgery registrar training programme, surgeons will achieve a Certificate of Completion of Training (CCT), which allows application to the GMC specialist register and the ability to apply for consultant position jobs.^16^ Each country has specific exit requirements, which are documented in Table 1. One requirement for the UK training programmes is completion of a logbook with development of independence in specific procedures, with completion of a certain number of procedures that the trainee has performed with or without supervision. A comparison of the emergency general surgical procedures required for CCT to demonstrate differences in the requirements at the end of training with the UK is made in Table 5.

**Table 4:** Comparison of second phase of general surgical training among 11 different countries*

Country	Name of programme	Years	Mandatory courses required?	Training all within general surgery?	Specialisation within general surgery?	Development of laparoscopic skills required?	Development of robotic skills required?	Endoscopy skills requirements
UK	General Surgery Specialty Registrar Training	6	ATLS Leadership course Teaching course	Yes	Yes – after year 4	Yes – competence required in selected surgeries dependent on sub-specialty	No	Only required for some sub-specialties; 200 colonoscopies required for colorectal surgery; 200 gastroscopies required for oesophagogastric surgery; 200 colonoscopies and 200 gastroscopies required for gastrointestinal and general surgery of childhood
India	Magister Churugiae (MCh), Surgical Gastroenterology (Note other MCh available in other general surgical sub-specialties)	2–3	Biostatistics Research methodology Laboratory medicine Techniques/courses relevant to management of gastrointestinal disease Use of computers/data science management in medicine Bioethics Ethical issues involved in management of gastrointestinal disease Hospital waste management Health economics	Yes	Yes – all training within sub-specialty	Yes – Insertion of laparoscopic ports, laparoscopic intracorporeal and extracorporeal suturing; Perform laparoscopic sleeve gastrectomy; Laparoscopic drainage of liver cyst or abscess	No	Yes – Independent in endoscopy; including endoscopic dilation, foreign body removal, clipping and glue techniques
Or	Doctorate of National Board – Surgical Gastroenterology	3	No	Yes	Yes – specialise in surgical gastroenterology – can carry out a further fellowship to sub-specialise further	Exposure to laparoscopic surgery	No	Yes – perform endoscopic procedures
Pakistan	Second Fellowship (colorectal surgery or HPB and liver transplant surgery or breast surgery)	3	Same requirements as for first fellowship	Yes	Yes – all training within a sub-specialty	Dependent upon hospital curriculum	No	Dependent upon hospital curriculum
Or	MS General Surgery	4	Typically no – but can depend upon curriculum	Yes	No	Yes – able to perform laparoscopic procedures (specific operations vary per curriculum)	No	Yes – can vary per curriculum Experience usually expected in diagnostic endoscopy and for upper gastrointestinal bleeding Colonoscopy and flexible sigmoidoscopy
Nigeria	General Surgery Residency training – part 2	4	Research methodology course	No – 3–3.5 years in general surgery with 6–12 months in other surgical specialty	No	Some experience in laparoscopic surgery, but no competencies required	No	Yes – 3 colonoscopy, 10 sigmoidoscopy
Egypt	Medical Doctorate (MD) in surgery	3	Depends on university curriculum – but often advise ATLS and laparoscopic courses	Yes	No	Depends upon university curriculum – but not routinely required	No	Depends upon university curriculum – but not routinely required
	Egyptian Board Certificate	5	Basic surgical skills Basics of laparoscopy BLS Trauma course Patient safety course Research methodology course Infection control Medical ethics Communication skills ICDL	No – early years spent time in other surgical specialties	No	Yes – competence in laparoscopic cholecystectomy	No	No
Ireland	General Surgery Specialty Registrar Training	6	ATLS Leadership course Teaching course	Yes	Yes – after year 4	Yes – competence required in selected surgeries dependent on sub-specialty	No	Only required for some sub-specialties; 200 colonoscopies required for colorectal surgery; 200 gastroscopies required for oesophagogastric surgery; 200 colonoscopies and 200 gastroscopies required for gastrointestinal and general surgery of childhood
Sudan	COSECSA fellowship	3	Research methodology Nonoperative technical skills for the surgeon (or similar leadership course)	Yes	No	Should have exposure to laparoscopic procedures	No	Perform flexible endoscopic procedures OGD with biopsy, colonoscopy and oesophageal dilation with supervision
	Medical doctorate (MD) in surgery –Part 2	3	No	Yes	No	Depends on curriculum – trainees report limited exposure	No	Depends on curriculum – trainees report limited exposure
Sri Lanka	Post MD training programme	3	No	Yes	Yes – all training with sub-specialty	Dependent on curriculum but generally yes	No	Yes – OGD and lower gastrointestinal endoscopy dependent on sub-specialty
Romania	General Surgical Residency	6	No	No – Mix of general surgery, vascular, urology, oncology, thoracic, ortho, plastic, neurosurgery, gynaecology, anaesthetics, emergency and bioethics	No	Yes – laparoscopic training module(3 months)	No	Participation in endoscopy
Iraq	General Surgical Training (information based upon the Arab Board Curriculum)		BLS Trauma course Emergency basic surgical skills Patient safety Research methodology	No – 18 months of general surgery, 3 months ED, 3 months ICU, 1 year in other surgical specialty – orthopaedics, urology, vascular, paediatrics, plastics, cardiothoracic, neuro; Fourth and fifth year in general surgery	No – a further fellowship in gastrointestinal surgery is possible with the Iraq board, limited information regarding this was found, however	Yes – can independently undertake laparoscopic cholecystectomy	No	No
South Africa	Registrar training numbered post + sub-specialty	3+2	ATLS	No – at least 3 months in ICU	Yes after 3 years	Know and understand the principles involved in laparoscopic surgery	No	Yes – be technically competent to perform diagnostic upper endoscopy and sigmoidoscopy

ATLS = Advances Trauma Life Support ; BLS = Basic Life Support; COSECSA = College of Surgeons of East, Central and Southern Africa; ED = emergency department; HPB = hepato-pancreato-billary; ICU = intensive care unit; OGD = oesophagogastroduodenoscopy

*Countries where there is only one phase of training have been included here

Some countries have separate programmes for sub-specialties of general surgery; when this occurs surgical gastroenterology has been chosen as the example, except for Pakistan where this is not a sub-specialty option. References used to collect data.^16,18,23–27,33,35–38,48,51,66–78^

**Table 5:** A comparison of the procedure numbers and demonstration of independence within emergency general surgery required for CCT within the UK for all trainees (except those who choose to undertake a pancreas transplantation or liver transplant and organ retrieval module or additional breast surgery module), with completion requirements for ten comparable countries

Country	Procedure and number requirement (supervisor trainer scrubbed at least)				
	Inguinal hernia	Cholecystectomy	Segmental colectomy	Emergency laparotomy	Appendectomy
UK	50	50	20	100	80
India	N/A	N/A	N/A	N/A	N/A
Pakistan	12	14	–	10	12
Nigeria	10	2	7	20	10
Egypt	15	16	2	10	15
Ireland	50	50	20	100	80
Sudan	N/A	N/A	N/A	N/A	N/A
Sri Lanka	10	7	5	4	10
Romania	8	5	4	8	30
Iraq	15	20	7	19	30
South Africa	N/A	N/A	N/A	N/A	N/A

CCT = Certificate of Completion of Training; N/A = Not available

Data have been collected from any single available curriculum within the country which has documented specific numbers (Appendix 1, available online).

## Discussion

Many variations are seen across the different countries with regards to postgraduate general surgical training. A difference is first seen in the number of years of experience working as a doctor prior to starting specialised general surgical training; this is a result of some medical degrees including an internship as part of the degree programme, such as India, some countries requiring experience in a range of surgical specialties prior to specialisation and some countries enforcing compulsory years of service.^31^ The UK has the largest requirement of time prior to specific general surgical training, requiring a minimum of 4 years of experience, compared with India and Romania where graduates can immediately begin general surgical training. As a result of this additional time and the requirement of compulsory sub-specialisation in general surgery, the UK has the longest path, requiring at least 10 years to qualify after medical school. The requirement of sub-specialisation without first qualifying as a specialist in general surgery also varies across countries, with most countries having the option to complete training by specialising just as general surgeon. In some countries a specific pathway for sub-specialisation was not available.

However, it is important to note that these are the minimum training times and not the average time for trainees to complete the programme. Competition to gain a position in training, success at examinations, breaks in training, completion of research components and fulfilment of the curriculum requirements, including gaining enough surgical experience, are all factors that can lengthen the time a trainee can take to complete the training process and these factors vary between individuals and countries.^27,52,53^ It is also important to note that although curricula for training programmes are often provided, these do not always match the reality of the training that trainees receive. In particular, this has been demonstrated in studies reviewing trainees’ experiences in laparoscopic surgery, which despite development of skills being included in surgical curricula, trainees report struggling to gain access and experience in.^67,79,80^ Within Sudan, it is also important to consider the effect their current political situation is having upon access to training, with many hospitals in the country currently being reported as non-functional because of the ongoing civil war.^81^

Although there are clear differences between countries in the length of time required to complete training, similarities were noted between training pathways, with all pathways requiring examinations to be sat and passed, and with oral, clinical and written components. For the majority of countries, a standardised curriculum for training could be found, and within these the requirement for a logbook to demonstrate progress was a common component. It is interesting to note that the number of operations trainees are required to have performed by the end of training is much higher within the training programmes for the UK and Ireland than in all of the other countries (Table 5). A possible reason for this difference is that the number of different documented surgeries in which independence is required is greater within the curricula of the other countries. In addition, experience is often also required in a greater variety of procedures, including procedures in the UK that would instead be carried out by other specialty surgeons, for example, vascular, urology and orthopaedic surgeons.

Although nearly all curricula included the development of laparoscopic skills, the amount of exposure does vary and, in some countries, can depend upon where in their country doctors are training, because of access to both equipment and trainers.^79,80^ The requirement for endoscopy skills to complete training also varies between countries, with some such as Egypt and Iraq not requiring any exposure. Currently no countries document any requirement for the development of robotic surgical skills despite its increased use in general surgery; it will therefore be interesting to see if this changes in the near future.

Variation also arose in the research requirements required for each training programme, with some countries requiring a thesis to achieve a degree such as a Master of Science (MS), a Doctor of Medicine (MD) or Master of Medicine (MMed), whereas others only require demonstration of evidence of experience in research.

### Study limitations

Although best efforts have been used to thoroughly research resources available for the general surgical training programmes across countries, it is important to note that for some countries difficulties were encountered because information was provided in a different language, particularly when accessing syllabuses and curricula. This required the use of research publications to gain some of the required information. It is also worth noting that most countries have alternative pathways for general surgical training, such as the UK Portfolio Pathway (formally known as CESR – Certificate of Eligibility for Specialist Registration), and although effort was made to try and detail well-described alternative pathways for countries, it is probable that some have not been included.^82^ It is also important to note that although this paper hopes to aid IMGs by explaining training programmes in their country, it will not necessarily be helpful for understanding the level of all IMGs from the ten countries described, because some IMGs who apply for general surgical positions in the UK will have not completed any training programme in their country, but instead have spent time working in surgical non-training jobs and may have instead worked towards gaining their Membership of the Royal College of Surgeons (MRCS).

## Conclusion

There are some similarities between the general surgical training programmes in the 11 countries listed including the requirement for completion of specific general surgical examinations. Many differences were also noted, particularly the length of time required to compete each training programme and the differing requirements for research. The information provided by this paper will hopefully increase the knowledge of general surgical seniors within the UK regarding the background experience of IMGs coming to the UK to work in general surgery, improving the ability to provide support for these doctors.
